# Diabetes and COVID-19: What we learned from the two ongoing pandemics

**DOI:** 10.1590/1518-8345.0000.3285

**Published:** 2021-10-29

**Authors:** Maria Teresa da Costa Gonçalves Torquato, Gil Cunha De Santis, Maria Lucia Zanetti

**Affiliations:** 1 Secretaria Municipal de Saúde de Ribeirão Preto, Programa de Aprimoramento Multiprofissional em Hipertensão Arterial e Diabetes Mellitus, Ribeirão Preto, SP, Brazil.; 2 Universidade de São Paulo, Faculdade Medicina de Ribeirão Preto, Hospital das Clínicas, Hemocentro, Ribeirão Preto, SP, Brazil.; 3 Universidade de São Paulo, Escola de Enfermagem de Ribeirão Preto, PAHO/WHO Collaborating Centre for Nursing Research Development, Ribeirão Preto, SP, Brazil.; 4 Universidade de São Paulo, Escola de Enfermagem de Ribeirão Preto, PAHO/WHO Collaborating Centre for Nursing Research Development, Revista Latino-Americana de Enfermagem, Ribeirão Preto, SP, Brazil.

**Figure:**
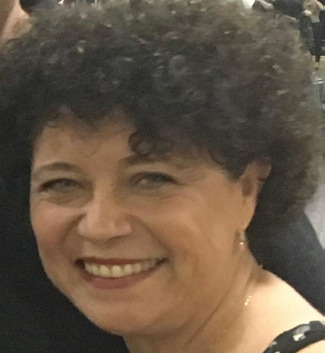


The pandemic due to COVID-19, an infection caused by the SARS-CoV-2 coronavirus initiated in Wuhan(China) in 2019, overlapped with a pre-existing pandemic, that of Type2 Diabetes Mellitus(DM2)^(1)^. The literature shows that DM2 represents a risk factor for the unfavorable evolution of COVID-19, as well as for contracting the disease, which means greater susceptibility to the virus. It was also shown that diabetic patients with good control presented more favorable evolutions when compared to those not controlled^(1-2)^.

**Figure:**
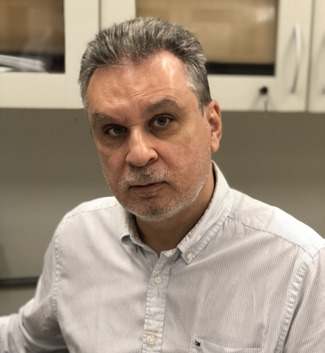


The UK Prospective Diabetes Study(UKPDS) showed that DM2 control prevents chronic complications^(3)^. In view of COVID-19, new knowledge reiterates the importance of controlling DM2, now focusing on preventing the severe form of an acute infectious disease.

**Figure:**
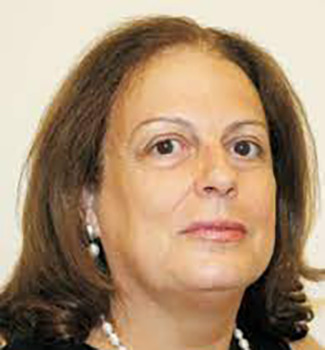


DM2 is defined by changes in glucose homeostasis and by a chronic inflammation condition, but it also induces changes in the immune system, making the patient affected by this disease more susceptible to infections, including those caused by viruses such as SARS-CoV-2. DM2 also causes activation of the renin-angiotensin-aldosterone system and endothelial damage, with a consequent increase in the risk of thrombosis. However, COVID-19 is a disease that can cause hyperinflammation and has been associated with an increased risk of occurrence of thromboembolic phenomena, especially pulmonary thromboembolism, more frequently observed in patients with severe pneumonia admitted to intensive care units(ICUs)^(4)^. The reasons for the association between DM2 and COVID-19 are as follows: lower functional reserve of organs caused by DM2, which can go unnoticed under normal circumstances, but comes to light as soon as a challenge of such magnitude as COVID-19 arises^(4)^. It is to be noted that the chronic inflammatory disease caused by DM2 is superimposed by the inflammation resulting from COVID-19, which then has its consequences greatly amplified.

In addition to the aforementioned aspects, DM2 is associated with other clinical conditions such as obesity, hypertension, coronary artery disease and chronic kidney disease, which are risk factors for the development of the severe form of COVID-19. Acute renal failure has been commonly observed in patients admitted to ICUs, especially in those with DM2^(4)^.

COVID-19 has caused significant sequelae, mainly in patients affected by the severe form of this disease, such as those who required mechanical ventilation. Apparently, sequelae are seen more frequently, and in a more severe form, in patients with DM2. The explanation would be linked to the lower functional reserve of the organs, in addition to an acute injury of greater magnitude, possibly caused by hyperinflammation. It remains to be determined whether the group of patients who survived COVID-19 will not have their DM2 condition worsened, as, in fact, seems to be the case. COVID-19 affects the pancreas, which could cause loss of beta cells, with possible worsening of DM2, for example, with the need to administer insulin, or require higher doses of oral antidiabetics. In addition, it does not seem unreasonable to us to assume that the diabetes complications may appear earlier and more severely.

In view of the COVID-19 pandemic, the health services initially closed their schedules for routine in-person appointments, recommending that patients with diabetes maintained social isolation, in addition to adopting other protective measures, such as use of masks and of alcohol gel. There was a need to restructure the services, organize schedules and the waiting room, regulate teleconsultation and train health professionals to provide safe care to patients with and without flu symptoms^(5)^. The pandemic has conclusively shown a chronic problem in the health services, that is, the need for multidisciplinary care for patients with diabetes.

Given the seasonality of viral diseases, vaccination against influenza was accelerated. Special attention must be given to the vaccination schedule of patients with DM2 for pneumonia^(5)^ and COVID-19 in accordance with the current protocols. It is to be noted that there is still no consensus on the tests to assess the immunization degree in people vaccinated for COVID-19^(6)^.

Considering the importance of good blood glucose control for lower susceptibility and better evolution of COVID-19 in people with diabetes, it is fundamental to intensify blood glucose monitoring for both asymptomatic individuals and more rigorously for those with flu symptoms or during an acute decompensation episode^(1-4)^ .

Healthy eating(avoiding ultra-processed foods, for example) is recommended^(2-5)^. Increasing consumption of industrially unprocessed vegetables, reducing the intake of food products characterized as fastfood and adopting habits such as adequate hydration should be encouraged. There are still no consistent data showing that dietary supplements can contribute to COVID-19 prevention and treatment.

Physical activity must be encouraged and adapted to the new context, as it regulates and strengthens the immune system of patients with diabetes, in addition to cardiovascular benefits and stress reduction^(2)^. Walking, reducing the time in a sitting position, practicing guided activities(in-person or virtually) outdoors, and adhering to the protocol for COVID-19 prevention can be considered safe measures. Performing lower limb and breathing exercises at home should be encouraged.

In relation to the COVID-19 diagnosis, it is important to reinforce the importance of not performing tests without a medical request and not taking medications without the guidance of a health professional; seeking early medical assistance in services structured to treat the disease; keeping prescribed medications to control diabetes; and remembering that therapeutic changes may be necessary.^(4)^.

Serious situations can require hospitalization, so that management of hospitalized patients with DM2, which was already a challenge before COVID-19, has had its importance intensified. Training of the multidisciplinary team and treatment customization are key aspects for the good evolution of the patient, with or without COVID-19. Adequate insulinization and reassessment of the prescription of oral medications, depending on the severity of the disease, increased this in-hospital challenge.

Patients with diabetes can present acute problems that require urgent care, for example, acute myocardial infarction or diabetic ketoacidosis, which must be early diagnosed and treated. It is necessary to advise the population not to be afraid to seek health services in the midst of the pandemic whenever they present symptoms suggestive of the aforementioned complications.

Given the complexity of the two pandemics, the patients must follow the health team’s recommendations and care. The messages conveyed by several types of media may not contribute scientific rigor(fake news) and veracity of the necessary information to define appropriate behaviors in the prevention and treatment of diabetes and COVID-19.

There are still several questions that could be answered in future studies, such as the consequences of the aggression to the pancreatic islet by COVID-19; whether there will be an increase in the incidence of DM1 and in the prevalence of DM2; and whether the pandemic will impose behavioral changes that favor greater control of chronic diseases such as diabetes.
